# Photoelastic Stress Analysis Surrounding Implant-Supported Prosthesis and Alveolar Ridge on Mandibular Overdentures

**DOI:** 10.1155/2010/780670

**Published:** 2010-05-10

**Authors:** Dorival Pedroso da Silva, Claudia Cazal, Fernanda Campos Sousa de Almeida, Reinaldo Brito e Dias, Rafael Yagüe Ballester

**Affiliations:** ^1^Departamento de Cirurgia, Prótese e Traumatologia Maxilo Faciais, Universidade de São Paulo, Av Prof. Lineu Prestes, 2227 - Cidade Universitária, São Paulo, 05508 000 São Paulo, Brazil; ^2^Departamento de Clínica e Odontologia Social, Curso de Odontologia da Universidade Federal da Paraíba, Brazil; ^3^Departamento de Odontologia Socia, Faculdade de Odontologia da Universidade de São Paulo, Brazil; ^4^Departamento de Materiais Dentários, Faculdade de Odontologia da Universidade de São Paulo, Brazil

## Abstract

The purpose of this research was to evaluate the maximum stress around osseointegrated implants and alveolar ridge, in a mandible with left partial resection through a photoelastic mandibular model. The first group consisted of two implants: traditional model (T), implants placed in the position of both canines; fulcrum model (F), implants placed in the position of left canine CL and right lateral incisor LiR. Both models linked through a bar and clips. The second group was consisted of three implants, with implants placed in the position of both canines (CR and CL) and the right lateral incisor (LiR), which composed four groups: (1) model with 3 “O” rings, (2) model 2 ERAs, bar with clips, (3) model 2 ERAs bar without clips; (4) model “O” ring bar and ERA. An axial and an oblique load of 6.8 kgf was applied on a overdenture at the 1st Pm, 2nd Pm, and 1st M. Results showed that the area around the left canine (CL) was practically free of stress; the left lateral incisor (LiL) developed only small tensions, and low stress in all the other cases; the right canine tooth suffered the largest concentrations of stress, mainly with the ERA retention mechanism.

## 1. Introduction

The prosthodontic rehabilitation of edentulous patients has been used over the years primarily for esthetics purposes after mandible resection. The development of osseointegrated implants allowed the production of more stable overdentures, improving retention and stability, thereby improving prosthetic rehabilitation prognosis and oral function. 

 Some researchers demonstrated the use of the photoelasticity to evaluate the stress of bodies subjected to efforts [1–4]. Other studies have exalted the success of the osseointegrated implants [5–8] pointing out their advantages in supporting overdentures. Some authors also [9] referred to the improvement of the dental prosthetic therapy with the use of two implants linked through a bar. Other fact, which must be considered, is the biting force, that increases three times more with the use of overdentures based on implants, when compared with conventional dentures. Some studies referred to the use of implant-supported prosthesis to compensate partially problems that arose from partial resection of the mandible [7, 8, 10]. 

 It is of great interest to know the tensions of the implants, also in cases of overdentures in patients with partial resection of mandible. Recent studies, using the method of photoelasticity [1, 2, 4, 11], have demonstrated the tensions in implant-supported prostheses, in which the largest tensions were found in the distal crests of the more distal implant. The obtained result, with the implant supported by overdentures, leads the authors to recommend the use of this technique with their patients with partial resection of mandible, but also suggested different retention mechanisms [2, 11] to the implants by using ball/O-ring and bar-clip attachments. They showed that ball/O-ring attachments transferred less stress to implants, than the bar-clip when the photoelastic model was subjected to a posterior vertical load. 

 The objective of this study was to evaluate, by the photoelastic method, the tensions around osseointegrated implants and ridge, used as overdentures support in patients with partial resection of jaw. 

## 2. Material and Methods

 A human jaw was duplicated and sectioned at the level of the left mental foramen. Three perforations corresponding to the 33 (CaL), 42 (LiR), and 43 (CaR) teeth were made in the model, where laboratory analog implants were installed, connected to molding cylinders, reproduced using silicone; the laboratory analogs were substituted by 13 mm implants (3I Implant Innovations, West Palm Beach, Fl.); by the use of photoelastic resin (PL-2; Photoelastic Division, Measurement Group, Raleigh, NC), the final model was obtained, and allowed the investigation of different retention mechanisms. Evermore, the evaluation of maximum tension was made in the left canine (CaL), right lateral incisor (LiR), right canine (CaR), and alveolar ridge below the overdenture. 

**Figure 1:**
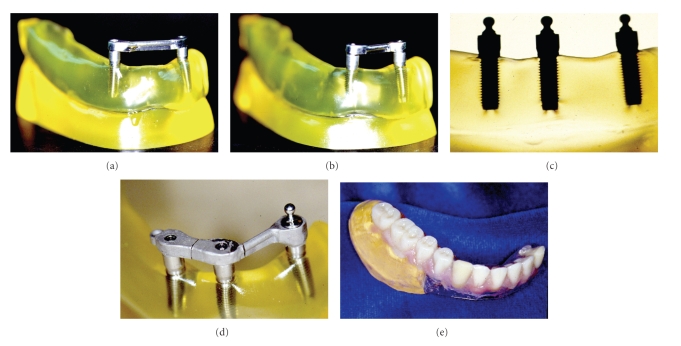
(a) Traditional model, (b) Fulcrun model, (c) Three implants with “O” rings, (d) “O” ring in the left canine, ERA in the right canine, linked with hader bar, (e) an example of overdenture.

 Six different groups of investigation were studied, with two implants: (1) T (traditional), implants in the positions of the canines, linked with Hader bar and yellow clips (Figure 1(a)); (2) F (fulcrum), implants in the positions of the left canine and right lateral incisor, linked with Hader bar and yellow clips (Figure 1(b)); with three implants, both canines and right lateral incisor: (3) Implants in the three positions with “O”  rings (Figure 1(c)); (4) two ERAs and clip, implants in the three positions with two ERAs in the canine teeth, linked for Hader bar and yellow clips; (5) two ERAs (without clip), similar to the anterior but without use of yellow clips; (6) retainer type “O”ring in the left canine and ERA in the right canine, linked with Hader bar and yellow clips (Figure 1(d)). 

 The overdentures were made by traditional method and fixed in position for the tests (Figure 1(e)). The applied load was 15 pounds (6.8 kgf) on the oclusal of 1st Pm, 2nd Pm, and 1st M surfaces. The direction of application was perpendicular to the occlusal plan and 15° inclined. 

The collected data were submitted to variance analysis. The values of the tensions in the implants were listed in five arrangements, (1) the support/group system, (2) the angle of incidence of the force, (3) the place of force incidence, (4) the place of the tension, and (5) the direction of the tension. The values corresponding to the tensions in the alveolar ridge consisted of 3 factors: (1) the support group, (2) the incidence angle, and (3) the incidence place. 

 The analyses were made separately for the tensions in the implant apex, and the alveolar ridge.

## 3. Results

The analysis of variance related to the tension on the implants, in the alveolar ridges and their arrangements with the various retention devices, showed variable statistical significant interaction. 

 The 22 experimental conditions showed statistical significant differences for the factor support/group system, but not for place or angle of incidence of the force. The interactions between the support/group system and the place of incidence of the force, and also between the support/group and the angle of incidence of the force, were also statistically significant (Table 1).

Table 1 presents the averages of the maximum tensions for the support/group system and their counterparts, as demonstrated by the Tukey test, and it also shows the averages amongst the tensions around implants for the six studied groups. Concerning the place and angle of incidence of the load, it was demonstrated that practically no group of the left canine tooth (CaL) presented stress. The stress was identified only on group six (“O” CaL) (ERA CaR) (CaL), and even so, with a very low value (0.07) and when the angle of load was inclined (0.10), when the place of the load was over the 1st Pm, in the right lateral incisor (LiR), only in group F the average value was relatively high (1.215 fringes/cm); even so it was smaller than in most other groups. In the right canine tooth (CaR), the concentration of stress in the groups with 2 ERAs was high, which reached 4.9 fringes/cm.

Table 2 values show that tensions in the ridge did not reach high values in any of the cases. It is also possible to observe that, as the load traveled further from the implants, the tension decreased in the implants and increased in the ridge, showing the negative correlation between tensions around implant and ridge. It was particularly notable that the total tensions in the implants and ridge were practically constant.

## 4. Discussion

Photoelastic methods have been applied to investigate biomechanical behavior of dental implants in bone supporting fixed and removable prostheses [12–14]; however, representing the nonhomogeneous and anisotropic structure of bone by plastic models gives rise to certain limitations in predictions of biological response to applied loads. Nevertheless, photoelastic models have successfully indicated differences between various conditions by comparative evaluation of stress-related outcomes.

 The Group T (traditional) presented the smallest tension at the alveolar ridge when compared to the other groups. The left canine of this group was the teeth with the lowest tension value (zero). In the right canine, the tensions did not get to be very high, despite being superior to the right lateral incisor of the Group F (fulcrum). Although tensions at the ridge have been larger in Group F than In Group T, it was smaller in the support element. Maybe this is due to the fact that the model T axes are parallel to the bar and to the ridges, and in the case of model F, axes are parallel only in relation to the remaining ridge. When there is application of load in the F model, even if the patient uses only one side during masticatory function, there is a slight rotation as in any normal expected overdenture and in the Model T, there is a decrease of this rotational regular functional and an unexpected torsion of the bar, causing increased load on implants and less load on the ridge.

 Small tensions in the implant are preferable than in the ridge. This protects the implant, whose loss would be clinically more problematic than an eventual reabsorption in the ridge [14].

 The lowest stress levels were observed for the Group 3 with “O” rings attachment system, which seems to present the more favorable clinical conditions showing a better distribution of tensions when the loads were uniformly distributed in ridge and also in the implants. This might mean that the ample covering of the basal area allowed for a better distribution of the load per unit area, as suggested by others [14].

 Both ERA retention groups, with and without clips, presented high stress levels mainly concentrated in the CR but not in the LiR and ridge, that maybe be linked to a larger setting in this group, which would be in agreement with authors [8] that found larger tensions in the distal implant when the load application was on elements in balance. 

 The group (“O” CaL) (ERA CaR) presented quite favorable results. Although the ridge suffers from a little high tension, the implants do not develop very high tension themselves. Kenney and Richards [16] and others [14, 16, 17] concluded that ball/O-ring attachments transferred less stress to implants than bar-clip attachments when the photoelastic model with 2 implants was subjected to a posterior vertical load. 

 Although several authors have associated implant-supported overdentures, as an indication for being used with patients without surgical resection problems [6, 7, 9, 12, 15], it becomes a valuable rehabilitation alternative in patients with partial resection of the jaw [5, 10]. 

 The negative correlation found between tension in the ridge and implants, “the more distant from the implants, the more is the load supported by the ridge”, offers an opportunity of therapy, even though temporarily. The fact is partly confirmed by the almost constant average stress in the implants and ridge. 

 Osseointegrated implants should be considered as a part of the mandibular rehabilitation plan of partially resected patient. Functional instability during aperture/closure movements might be partially established by implants and overdentures, even though they do not achieve all renowned biomechanical aspects. The aim is to minimize stability problems which may emerge with an “O” ring retention or Bar retention mechanism. Aspects related to irradiated patient might be considered cautiously such as radiation dosage and exact local. 

 We may also consider that patient-mediated factors such as retention, jaw morphology, jaw physiology, and unfortunately, financial considerations dictate the number and design of implants and the design of the mandibular overdenture prosthesis.

## 5. Conclusions

Within drawn methodology conclusions were presented in the following.

1. In most cases, very low stress level was transferred to the left canine next to the mandible resection.
2. The contralateral canine (next to the prosthesis) accumulated the highest tensions, especially those of the both ERA groups.
3. The more distalized, the application of the load resulted in decreased stress transfer to the implant and increased to the alveolar ridge.
4. Considering the group of three implants, the group 06 (“O” CaL) (ERA CaR) presented the smallest concentrations of tensions in the right canine tooth.

## Figures and Tables

**Table 1:** Averages of the relative tensions (fringes/cm number) in the apex of the implants, corresponding to the interactions, support/group system × angle of incidence of the force (*n* = 18), and support/group system × place of incidence of the force (*n* = 12).

	Support/Group	Angle of Load*		Place of Load**		
		Perpendicular	Inclined	1st Pm	2nd Pm	1st M
01	T (CaL)	0.00	0.00	0.00	0.00	0.00
02	T (CaR)	1.84	1.08	1.93	1.35	1.09

03	F (CaL)	0.00	0.00	0.00	0.00	0.00
04	F (LiR)	0.87	1.56	1.41	1.25	0.99

05	3 “O” (CaL)	0.00	0.00	0.00	0.00	0.00
06	3 “O” (LiR)	0.00	0.76	0.57	0.31	0.26
07	3 “O” (CaR)	1.60	2.50	2.24	1.77	2.14

08	2 ERAs + clip (CaL)	0.00	0.00	0.00	0.00	0.00
09	2 ERAs + clip (LiR)	0.17	0.59	0.57	0.37	0.21
10	2 ERAs + clip (CaR)	1.60	4.86	3.65	3.18	2.87

11	2 ERAs no clip (CaL)	0.00	0.00	0.00	0.00	0.00
12	2 ERAs no clip (LiR)	0.10	0.52	0.37	0.31	0.26
13	2 ERAs no clip (CaR)	1.98	4.90	4.01	3.54	2.76

14	(“O” CaL) (ERA CaR) (CaL)	0.00	0.07	0.10	0.00	0.00
15	(“O” CaL) (ERA CaR) (LiR)	0.14	0.73	0.52	0.47	0.31
16	(“O” CaL) (ERA CaR) (CaR)	2.57	0.80	1.98	1.77	1.30

*Tukey (5%) = 0.859 (interaction support/group system × angle of incidence of the force). Letters for comparisons a to i, when same averages are similar.

**Tukey (5%) = 1.107 (interaction support/group system × place of incidence of the force). Letters for comparisons k to v, excluding m, when same averages are similar.

**Table 2:** Averages of the relative tensions (fringes/cm number) in the edges of the implants, corresponding to the interactions, system apparel (implant system Type) × angle of incidence of the force (*n* = 03) and system apparel × place of incidence of the force (*n* = 02).

	Support/Group	Angle of Load*		Place of Load**		
		Perpendicular	Inclined	1st Pm	2nd Pm	1st M
01	T (Ridge)	0.63	0.83	0.31	0.94	0.94
02	F (Ridge)	2.50	1.04	1.56	1.88	1.88
03	3 “O” (Ridge)	1.25	1.25	0.63	1.25	1.88
04	2 ERAs + clip (Ridge)	1.25	1.25	0.94	0.94	1.88
05	2 ERAs no clip (Ridge)	1.25	1.46	0.94	1.25	1.88
06	(“O” CaL) (ERA CaR) (Ridge)	1.46	2.29	1.88	1.88	1.88

*Tukey (5%) = 1.205 (interaction system apparel × angle of incidence of the force). Letters for comparisons a to c, when same, averages are similar.

**Tukey (5%) = 1.604 (interaction system apparel × place of incidence of the force). Letter for comparison d, when equal, averages are similar.

## References

[1] Cehreli M, Duyck J, De Cooman M, Puers R, Naert I (2004). Implant design and interface force transfer. A photoelastic and strain-gauge analysis. *Clinical Oral Implants Research*.

[2] Kenney R, Richards MW (1998). Photoelastic stress patterns produced by implant-retained overdentures. *The Journal of Prosthetic Dentistry*.

[3] Thayer HH, Caputo AA (1980). Photoelastic stress analysis of overdenture attachments. *The Journal of Prosthetic Dentistry*.

[4] White SN, Caputo AA, Anderkvist T (1994). Effect of cantilever length on stress transfer by implant-supported prostheses. *The Journal of Prosthetic Dentistry*.

[5] Ekstrand K, Kjellman O (1986). The resected edentulous mandible - prosthetic treatment with implant-fixed prosthetis: a new clinical technique. *Gerodontics*.

[6] Lewis S (1993). Implant-retained overdentures. *Compendium of Continuing Education in Dentistry*.

[7] Misch CE, Misch CE (1996). Opções de tratamento para overdenture mandibular implantada. *Implante Odontológico Contemporâneo*.

[8] Zarb GA, Jansson T, Jemt T, Branemark PI, Zarb GA (1985). Other prosthodontic applications. *Tissue Integrated Prostheses: Osseointegration in Clinical Dentistry*.

[9] Setz J, Kramer A, Benzing U, Weber H (1989). Complete dentures fixed on dental implants: chewing patterns and implant stress. *The International Journal of Oral & Maxillofacial Implants*.

[10] Cilento PA, Nimmo A (1992). Use of implants to restore dentition in a partially resected mandible: clinical report. *Implant Dentistry*.

[11] Ueda C, Markarian RA, Sendyk CL, Lagana DC (2004). Photoelastic analysis of stress distribution on parallel and angled implants after installation of fixed prostheses. *Pesquisa Odontologica Brasileira*.

[12] Akça K, Fanuscu MI, Caputo AA (2008). Effect of compromised cortical bone on implant load distribution. *Journal of Prosthodontics*.

[13] Sadowsky SJ, Caputo AA (2004). Stress transfer of four mandibular implant overdenture cantilever designs. *Journal of Prosthetic Dentistry*.

[14] Costa MM, da Silva MAMR, Oliveira SAG, Gomes VL, Carvalho PM, Lucas BL (2009). Photoelastic study of the support structures of distal-extension removable partial dentures. *Journal of Prosthodontics*.

[15] Meng TR, Rugh JD (1983). Biting forces in overdenture and conventional denture patients. *Journal of Dental Research*.

[16] Kenney R, Richards MW (1998). Photoelastic stress patterns produced by implant-retained overdentures. *The Journal of Prosthetic Dentistry*.

[17] Celik G, Uludag B (2007). Photoelastic stress analysis of various retention mechanisms on 3-implant-retained mandibular overdentures. *Journal of Prosthetic Dentistry*.

